# Making of a pediatric cardiac surgeon, in India

**DOI:** 10.4103/0974-2069.41056

**Published:** 2008

**Authors:** Rajesh Sharma

**Affiliations:** Department of Pediatric and Congenital Heart Surgery, Escorts Heart Institute and Research Centre, New Delhi, India

## A. ANOTHER DAY AT THE OFFICE

DAY 1: He couldn't help kicking himself for missing the obvious. It was 12 hours since the baby had been taken into the OR for an arterial switch. The operation was long over. Yet here he was still scrubbing in for the same case, half a day later. The child had been shifted back to OR for bleeding-30-40 ml /hr - enough for a neonate to not maintain pressures and enough to demand continuous blood transfusion that was now beginning to drop the pO2. Chest open, the culprit was the suture line of the left coronary button at 5'o'clock position towards him. Where the inverting suture line of the button transitions into an “everting one” a small dog-ear inevitably forms underneath the newly constructed pericardial patch of the main pulmonary artery. Not a good place to try placing a suture without cardiopulmonary bypass. In this 2 mm of space, if a needle that is inserted into the aorta is not picked up from the coronary button in one stroke, a trickle could become a river and he would forever regret the misadventure. He put in a sliver of surgicel and decided to wait. It stopped. As he scrubbed out, none other than he knew how close they had been to disaster. From then on in every coronary button implantation, at that point of transition, a reinforcing mattress suture would be placed prophylactically.

Bleeding is a dreaded complication after the arterial switch operation. The problem is that the pulmonary artery circumferential suture line hides the posteriorly placed aortic suture line. In trying to look for suspected bleeding once the surgery is over, you run the risk of producing bleeding where none may have been present. Hence the role of preventing it.

√ Use a smaller needle.

√ Drag in the adventitia with each stitch.

√ Prophylactic additional sutures on every perceived gap.

…….The checklist keeps increasing.

DAY 2: He was driving out with his family on a weekend. Two hours out of the city he received a call from the hospital. A child he had operated for tetralogy of Fallot ten days back was cold and clammy and shifted back to ICU. He had seen the child in the morning on the rounds in the ward. There had been no problem. Only, for the last few days he had been complaining of chest pain, despite adequate analgesics. Something children do not complain even after a sternotomy. The chest X-ray and echo were normal. So he had not pursued it. The size of pulmonary arteries had been borderline and therefore the RV/LV pressure at the end of the surgery was high-0.8. But had otherwise done well and was to be discharged. Then came a second call. The child was intubated and CPR instituted. Third call. Blood pouring from the drain sites. Chest opened by a colleague. Finding: Dehisced RVOT patch.

MORAL : Continuous suture lines need to be reinforced at the end of the procedure before coming off bypass.

The child, in his own way, had told him about the problem many days ago when he complained of chest pain. He, the treating surgeon, had failed to understand the hidden message.

DAY 3: Conduit placements: He had been doing them for more than a decade as pulmonary artery substitutes for pulmonary atresia. Why were some going home with a systolic murmur and a gradient of 20 mm Hg at the take off from the RV despite no obvious technical reason? Watching the result after one such operation before scrubbing out, it was suddenly clear why. The left and right-sided conduit edges that were being sutured to the ventriculotomy edge were being prepared symmetrically. In the normal position, with the heart pointing leftward, any vertical right ventriculotomy also points leftward as does the left pulmonary artery to which the distal sutured edge of the left side of the conduit leads. Perhaps the proximal end of the conduit hood needs to be cut asymmetrically, with more length being given to the rightward edge than to the left. The next Rastelli, the proximal conduit hood was fashioned with the leftward aspect cut parallel to the conduit annulus and the right ward obliquely. Result: A perfect lie. No gradients at discharge or on follow up since.

All the above examples describe defining moments of an operation: Hemostasis after an arterial switch operation, prevention of secondary hemorrhage from a high-pressure suture line, or constructing a conduit devoid of obstruction. All have serious implications for the patient. Yet, once the problem has been analyzed, the solutions are very simple and down to earth. Surgery, in general, is no frills - very basic. It only demands that you pay attention to the physics that a particular reconstruction demands. Once that is taken care of, little else is required.

The list of such learning sessions is never ending.

Learning in Pediatric cardiac surgery is a continuum. In the eyes of the outside world, training gets over when a surgeon becomes an independent consultant. In reality he remains a trainee forever.

The quest to achieve perfection is infinite. In a subject as diverse as correction of congenital heart defects, this implies that given the large number of heart conditions, you would need a hard disc with an infinite number of gigabytes to document the experience of an active congenital cardiac surgeon. In a path traveled everyday with high power magnification in a cylinder of vision limited to the cone of light originating from the headlight for hours on end, everyday, in a quest to better the performance of the previous day, it is indeed frightening where this goal of the ‘perfect’ result leads one to.

Till you achieve perfection you remain, to yourself, a trainee. Only, ‘perfection’ is a relative word.

## B. PAEDIATRIC CARDIAC SURGERY – THE INDIAN SCENARIO

India's is a young population. The country has arguably the highest birth rate in the world. The heart is the organ most commonly afflicted by congenital lesions. Yet India has a huge dearth of pediatric cardiac surgeons. While we could do with more adult cardiac surgeons too, the shortfall of pediatric cardiac surgeons in the country is so acute that it gives the adult group an impression of relative surfeit.

At a time when the supply of patients to the adult cardiac surgeon is dwindling secondary to competition from interventional cardiology colleagues, adult cardiac surgery still continues to attract young blood into its fold.

The reasons for this skewed ratio of adult to pediatric services in cardiac surgery are not very difficult to fathom. Adult cardiac surgery is more finite, more predictable, caters to the breadwinner of the family, who, not uncommonly, occupies a prominent position in society. The child, more often than not, hails from a family of average means. Operations are arduous and much more demanding, and are followed by the necessary recovery period in the intensive care unit, which can be very intimidating to the most stout hearted surgeon. Prolonged hospital stays are not uncommon and hence likely to be less profitable, even a financial loss, for the hospital management.

Despite all of the above, there is a resurgence of interest among younger generations of cardiac surgeons. Highly competitive adult practice is a likely etiology. The rapidly increasing burden of congenital heart disease in the country is increasing the demand for more hospitals to start pediatric cardiac surgery units. More employment opportunities will definitely encourage young trainees to take up this profession.

In today's nuclear families, the child occupies an important position in a family. Parents are putting a higher premium on the wellbeing of their children. Another reason for the renewed interest in this speciality could also be the fact that today, unlike olden days, there are qualified colleagues in different aspects of pediatric cardiac care who will shoulder the responsibility of getting a child through after a heart operation. A surgeon in India today can limit himself more to the operation and let other professionals sort out non-surgical medical issues.

Or can he, really?

Most cardiac centers in the country today still have no dedicated intensivist to manage the surgical ICU. The surgeon quite often has to double as the intensive care specialist. He needs to operate upon, and also take care of his patient - manage ventilatory support, judge inotrope requirement, manage infections, assess the importance of residual lesions. In other words he needs to be a veritable one man army. But as solace to the aspiring, responsibilities are now getting shared, and compared to the past, the situation is rapidly improving. Younger postgraduates are joining specialties like critical care medicine devoted to children and are ready to be trained to take up position alongside him.

In most pediatric cardiac centers of the country today, the buck starts with and also stops at the surgeon. To be able to select, conduct, and evaluate an operation for cardiac conditions on a regular, day-to-day basis, he needs to live, breath and dream pediatric cardiac surgery. Most functional congenital cardiac programs in the country are built around competent surgeons and pediatric cardiologists. These individuals were not born overnight. They are the result of decades of evolution with many successes and failures serving as the foundation.

### OPERATING UPON THE CHILD'S HEART

The variations that embryogenesis can bestow upon the heart are mind-boggling. They occur at all levels -the inlet, the outlet, the inside, and can be in the form of abnormal connections, narrowed pathways, holes etc. In this kaleidoscope of lesions, you can imagine any abnormality and it is there. A child's heart is truly fascinating in the ways it can challenge a surgeon to bring it back to normalcy.

The baby born with critical congenital heart disease is a strict taskmaster. Ventricular septal defect closure needs to be watertight. And there better be no obstruction to the outflow tracts of the ventricles. In a third world set up, in the absence of specialized help, a residual defect has a high chance of resulting in a major morbidity or even mortality. To achieve this end, a flawless surgical repair is to be pursued. The end result needs to be checked out before leaving the operating room (step up in O2 sat between right atrium and pulmonary artery) to evaluate ventricular septation, direct pressure measurements to look for residual gradients, TEE, or epicardial echo.

In addition to settling anatomic issues, cardiac function should be preserved, as also the function of other parts of the body, notably the kidneys and the brain. Perfusion-related issues like hematocrit, perfusion pressure, line pressure, venous drainage, adequacy of flows, rate of cooling and re-warming are all finally the responsibility of the surgeon.

### AN ENGINEER WITHOUT A DEGREE IN ENGINEERING

In all fairness to the patient (or the underlying lesion), outcomes after a congenital cardiac repair are reasonably predictable. If the result is at variance, it is a lack of understanding that is the likely cause, as inevitably some aspect of the problem would have been ignored or not paid as much attention as it deserved or an issue of operability.

A congenital cardiac repair is as three dimensional as the heart itself. A surgeon dealing with the heart is nothing short of a mechanical engineer who has to assess the length, breadth, curvature, convexity or concavity of a bridge or a girder that has to bridge two fixed (or moving) structures. In addition he has to ensure that what he creates stands the test not only the test of the vigorous cardiac motion but also the test of time. In fact he has to provide for something that no engineer has to wrack his brains about: growth. What he creates today needs to stand the heart in good stead for a lifetime.

Examples of repairs that challenge the skill and the imagination of every surgeon dealing with them include prominently, intraventricular tunnel for double outlet right ventricle, complex coronary transfer, repair of atrio-ventricular canal defect, the venous baffles of the atrial switch, to name a few.

“Pediatric cardiac surgery separates men from the boys”-Dr Chris Barnard.

### SURGICAL TRAINING

Does cardiac surgical training available in India today make one upto all of the above? As per the curriculum, the “would-be” cardiothoracic surgeon needs to get selected into a recognized course offering either MCh or a DNB (Cardiothoracic Surgery Board equivalent) in cardiothoracic surgery, after completing his masters in General Surgery. In both the MCh and the DNB, stress would be on handling the adult cardiac patient. Pediatric cardiac surgery does form a good bulk of most teaching institutions offering these courses today (more so the MCh course), but it is hardly sufficient to prepare the candidate to stand alone in the complex world of pediatric cardiac surgery. Even in adult cardiac surgery, hands on surgical experience in managing a case independently would be fairly limited. This is in contrast to, say, a cardiac surgery training in the US, where each resident in cardiothoracic surgery needs to log in a minimum number of operations independently. By the time he exits from the program, he is reasonably proficient in handling the bread and butter of cardiac surgery, i.e., straightforward coronary bypass, valve replacement, etc. A program that does not permit the student enough surgery runs the risk of de-recognition.. In India, training is more didactic rather than hands-on. Independent handling of operations by trainees is an exception. The result is that even in adult cardiac surgery, you have an MCh degree but still need further experience to gain the confidence to be able to operate independently.

### 36 CHAMBERS OF SHAOLIN?

Real learning even in adult cardiac surgery starts only once you are a consultant with some degree of responsibility and to expect a recently qualified MCh or DNB to handle pediatric cardiac surgery would be completely impractical. The actual molding of a Pediatric cardiac surgeon starts in the gurukul of his mentor who he joins as an apprentice. It is here that with each passing day and case, he learns every minute aspect of pediatric cardiac care, the rough stone starts to be chiseled out. As time elapses, certain definite signs of reigning in of the overenthusiastic, hypereflexic fresher start becoming evident. Perhaps the first important feature is the perceptible reduction in rapid, jerky movements that are so common in the young trainee fresh out of the adult world. Movements become slow and deliberate, the power of concentration increases with increase in attention span. The apprentice stops to crave for speed and focuses on accuracy. As accuracy becomes second nature, the number of attempts to achieve each step comes down. Without trying to be fast, speed increases.

Attitude is another aspect of a pediatric cardiac surgeon in the making. The attitude: “Learn at all cost” needs to take precedence over all other aspects of existence. This attitude is not always conducive to comfortable existence, neither for the trainee's ego nor his or his dependents' circadian rhythms. But this is how the rock gets hewn. If he and his people can stand this training by fire they have a priceless diamond in the making. The apprenticeship is long and painstaking. It is here that the veritable “cloning” occurs. The apprentice starts to turn into a copy of his teacher. Once he is proficient enough, in later life, he will automatically modify and improve upon it with his growing experience and perhaps with inputs from other sources and carve out a style of his own.

### TRAINING ABROAD

In bygone days, there was no other place to train in pediatric cardiac surgery than, say, in Buffalo Children's or Boston Children's. Today, operations of all types and complexity are being performed in the country. Training in any of these places can benefit the discerning mind way beyond the learning expectations abroad. The sheer number of cases provides an experience unmatched in any other place the world over.

So does that imply that the foreign experience is a waste of time?

Definitely not! The role of experience in foreign centers today, to my mind, is more after one is already a full fledged surgeon - in picking up newer variations, in imbibing different techniques and improving oneself.

### FEEDBACK LOOPS

Another extremely important aspect that a young would-be pediatric cardiac surgeon has to learn and retain as he grows is to retain a robust feedback mechanism. The quality to introspect should stay with him till the last day of his active surgical life. No adverse event occurs without a reason-and a physical reason at that-needs to be remembered. In a busy surgical practice, any problem that occurs once is bound to recur unless the cause is properly dissected and a preventive mechanism instituted. An optimum surgical repair is often dependent on simple physics (that is synonymous with common sense) and anybody's and everybody's viewpoint is relevant where common sense plays such a major role.

Dr Denton Cooley is quoted to have said; “Nothing in my life has challenged me more than the repair of a baby's heart”.

“If you had the chance to commence your cardiac surgical career all over again, which branch would you select?” Pat came the reply: “Pediatric cardiac surgery”.

